# Frontal, Sensorimotor, and Posterior Parietal Regions Are Involved in Dual-Task Walking After Stroke

**DOI:** 10.3389/fneur.2022.904145

**Published:** 2022-06-22

**Authors:** Shannon B. Lim, Sue Peters, Chieh-ling Yang, Lara A. Boyd, Teresa Liu-Ambrose, Janice J. Eng

**Affiliations:** ^1^Department of Physical Therapy, University of British Columbia, Vancouver, BC, Canada; ^2^Rehabilitation Research Program, GF Strong Rehabilitation Centre, Vancouver, BC, Canada; ^3^School of Physical Therapy, Western University, London, ON, Canada; ^4^Department of Occupational Therapy and Graduate Institute of Behavioral Sciences, College of Medicine, Chang Gung University, Taoyuan City, Taiwan; ^5^Department of Physical Medicine and Rehabilitation, Chang Gung Memorial Hospital, Chiayi, Taiwan; ^6^The David Mowafaghian Centre for Brain Health, University of British Columbia, Vancouver, BC, Canada; ^7^Centre for Hip Health and Mobility, Vancouver Coastal Health Research Institute, Vancouver, BC, Canada

**Keywords:** functional near-infrared spectroscopy, stroke, gait, dual-task, posterior parietal cortex (PPC), sensorimotor cortex (SMC), pre-motor cortex (PMC), prefrontal cortex (PFC)

## Abstract

**Background:**

Walking within the community requires the ability to walk while simultaneously completing other tasks. After a stroke, completing an additional task while walking is significantly impaired, and it is unclear how the functional activity of the brain may impact this.

**Methods:**

Twenty individual in the chronic stage post-stroke participated in this study. Functional near-infrared spectroscopy (fNIRS) was used to measure prefrontal, pre-motor, sensorimotor, and posterior parietal cortices during walking and walking while completing secondary verbal tasks of varying difficulty. Changes in brain activity during these tasks were measured and relationships were accessed between brain activation changes and cognitive or motor abilities.

**Results:**

Significantly larger activations were found for prefrontal, pre-motor, and posterior parietal cortices during dual-task walking. Increasing dual-task walking challenge did not result in an increase in brain activation in these regions. Higher general cognition related to lower increases in activation during the easier dual-task. With the harder dual-task, a trend was also found for higher activation and less motor impairment.

**Conclusions:**

This is the first study to show that executive function, motor preparation/planning, and sensorimotor integration areas are all important for dual-task walking post-stroke. A lack of further brain activation increase with increasing challenge suggests a point at which a trade-off between brain activation and performance occurs. Further research is needed to determine if training would result in further increases in brain activity or improved performance.

## Introduction

Successfully walking within the community requires an intricate ability to continue walking while completing various additional tasks such as conversing and avoiding obstacles (i.e., dual-tasking). The addition of these secondary tasks often results in a decline in performance compared to completing the task on its own (i.e., walking only or talking only). After a stroke, the magnitude of decline is typically significantly larger than that observed in their age-matched healthy counterparts (1) and the decline in the overall dual-task performance are related to an individual's functional ambulation category (2) and number of falls (3). In order to improve walking post-stroke, it may be important to understand the mechanisms underlying dual-tasking in this population.

Performance of two tasks simultaneously may require additional or alternative neural resources compared to completing each task on its own (4). Several mechanistic hypotheses have been proposed to help explain performance during dual-task walking. Depending on the neuronal requirements of each task, there can be a bottleneck (5), lack of resources (6), or capacity limit (7) that impact the performance of each task when completed together. In brief, the Bottleneck Theory posits that certain components of each task are able to undergo parallel processing, however, if the two tasks require similar processes at the same time [e.g., response selection (8)], a bottleneck occurs and only one task will be completed at a time (5). Therefore, when asked to simultaneously complete two tasks (e.g., walking and arithmetic), if one task requires longer processing (e.g., arithmetic), the completion of the second task will be delayed (e.g., decreased gait speed). The Multiple Resource Theory suggests that there is a limit on the number of resources available at a given time. If two tasks require more resources than what is available, especially similar resources, a deterioration of one or both tasks may be observed (9). Theoretically, simultaneous completion of two motor tasks would have greater interference than completing a cognitive and motor task simultaneously. Finally, the Capacity Limit Theory states that parallel processing can occur when two tasks are presented, however, functional resources will be shared between the two, resulting in an increased completion time for both tasks (7). For a single task, in comparison to healthy adults, individuals with stroke generally show greater brain activity (10). With this already elevated brain activation, a limit may be met sooner in individuals post-stroke. Several studies have shown this capacity limit in older adults where neural activation no longer increases or sometimes even decreases with dual-tasks [review: (11)].

Evidence of this across multiple cortical regions in the stroke population is limited. After stroke, we have shown that faster walking is related to an increased magnitude of brain activation in prefrontal cortex (PFC), sensorimotor cortex (SMC), and posterior parietal cortex (PPC) (12). When walking complexity increases, increased activation has primarily been observed in PFC and only a few studies showed activation in pre-motor cortex (PMC) and SMC [review: (13, 14)]. While the PFC plays an important role in dual-task walking, the literature has also identified other cortical areas that are involved in dual-tasking [review: (4)]. These areas include the PMC (15, 16), SMC (17, 18), and PPC (19, 20). The functional role of these additional areas can provide some insight on how resources are being allocated during different walking complexities. For example, increases in PMC activity would suggest a need for more motor planning and preparation, SMC increases would suggest an impact on motor output or sensory input, and PPC activity would suggest heightened need for sensorimotor integration. Additionally, the relationship between brain activation and clinical measures is unclear; one study showed that higher cognitive and motor status predicted greater brain activation during complex walking (21) but another study showed a negative relationship with motor status and dual-task brain activation (22). Thus, further work is needed to characterize brain activation during dual-task walking after stroke and to determine the relationship between the activation levels and clinical measures.

The overall purpose of this study is to investigate how brain activation changes with dual-task walking after stroke and how it relates to an individual's cognitive and motor status. Specifically, we first look at how the addition of a secondary cognitive task to an existing motor task will increase overall brain activation and if the addition of a more difficult secondary cognitive task will further increase activation. Secondly, we aim to determine if the magnitude of regional brain activity changes relate to an individual's cognitive or motor status.

## Methods

### Participants

#### Recruitment

Participants were recruited by purposive sampling through posters at local rehabilitation centers, private clinics, and online platforms. Study details were also disseminated through phone or mail to previous participants who have agreed to be contacted for future studies. The study was approved by the university clinical research ethics board and all participants provided written and informed consent.

#### Screening

Interested individuals were first screened for eligibility *via* telephone. Inclusion criteria included an age ≥18, telephone Mini-Mental Status Exam >21/26 (23, 24) indicating mild cognitive impairment at most, stroke incident >6 months previous (i.e., chronic stroke), one-sided hemiparesis, able to walk independently (gait aids allowed) for 1-min bouts, able to understand and follow directions in English, and able to clearly communicate verbally. Exclusion criteria included orthopedic injury impairing current walking, neurological injury other than stroke, and multiple known strokes.

#### Demographic Data

Age, sex, and gait aid used were collected. When available, stroke characteristics were obtained through medical charts. When charts were not available, details on time post-stroke, and stroke type (ischemic/hemorrhagic) were collected through verbal reports by the participants. Lesion location was determined through structural MRI obtained through medical records or collected for this study when eligible.

### Task Procedure

**Figure 1:**
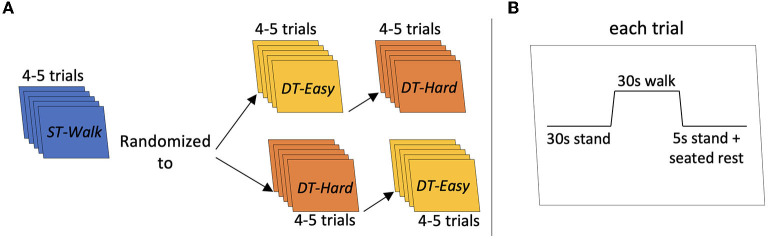
Schematic of task procedure. **(A)** Indicates the procedure for each trial. **(B)** Indicates the order of the conditions. Each participant started with 4–5 trials of *ST-Walk first* then they were randomized to either *DT-Easy* or *DT-Hard* walking conditions next.

The walking tasks were completed in a 50-m hallway. Participants performed three walking conditions: walking only (*ST-Walk*), walking while saying a word repeatedly (*DT-Easy*), and walking while completing a verbal fluency task (*DT-Hard*). All participants first completed the *ST-Walk* condition then either the *DT-Easy* or *DT-Hard* condition (randomized) (Figure 1A). For the *ST-Walk* condition, participants were asked to walk at their comfortable-pace. During the *DT-Easy* condition, participants were told to walk while continuously and audibly saying one of the following words: ma, pa, da, ba, ta. Finally, for the *DT-Hard* condition, participants were told to walk while saying as many words as possible that started with one of the following letters: B, R, D, C, H. Repeat words, proper names, or words with similar prefixes but a different suffix were not accepted. These specific letters were classified as easy-moderate difficulty (25) and has approximately the same number of words within the English Oxford dictionary.

Participants were first provided with 1–2 familiarization trials of each walking condition. Each trial started with the participant standing at either end of the hallway. The starting side was randomly determined by the researcher. After a minimum 30 s quiet stance, a verbal “go” from the researcher indicated the start of the walking trial and a verbal “stop” indicated the end of the trial. All participants were given instructions to keep their head position consistent and avoid talking throughout the walking only trials. Each walking trial was 30 s long and was performed 4–5 times. For all trials, a manual wheelchair and a spotter were positioned behind the participant for safety. At the end of each walking trial, participants stood for 5 s; they were then asked to sit in the wheelchair and then pushed to the end of the hallway to start the next trial. All trials had at least 30 s of quiet standing immediately prior to the start of the trial (Figure 1B)—this allowed for the functional near-infrared spectroscopy signals to return to baseline and a portion of this period was used for baseline comparisons. The PychoPy3.0 program was used for randomizing the conditions and triggering/timing the trials (26).

### Measures

#### Functional Brain Activation

**Figure 2:**
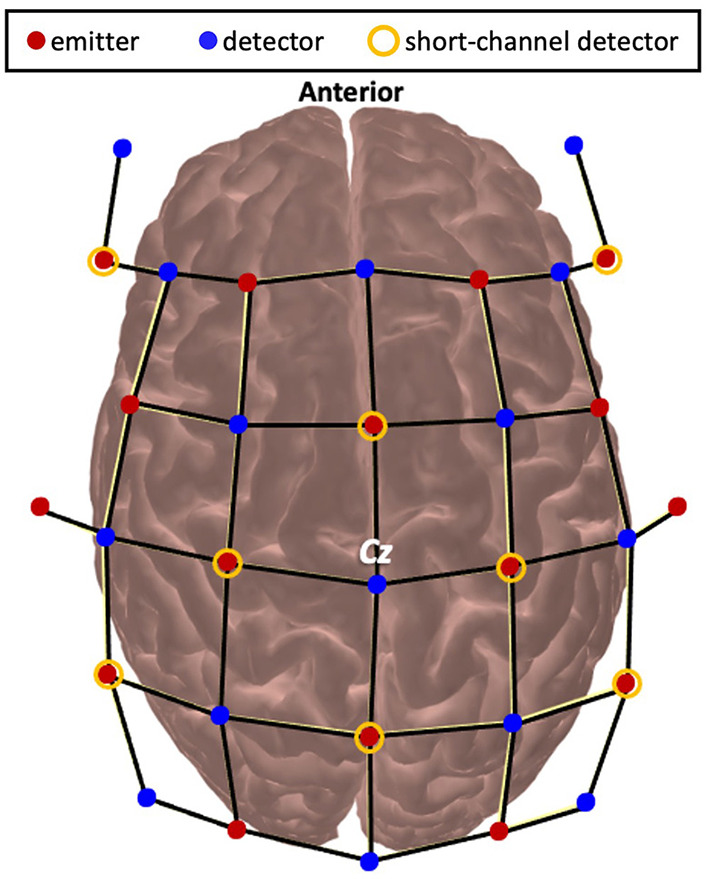
Montage of optode placement over the scalp.

Functional brain activity was measured using functional near-infrared spectroscopy (fNIRS). A wireless and portable fNIRS device (NIRSport2, NIRx Medical Technology, Germany) was used. Emitters released near-infrared light at 760 and 850 nm which enabled measurement of both HbO and HbR. The optodes were wired to the fNIRS collection device that was worn as a backpack by the participants. fNIRS data were continuously sampled at 4.36 Hz through Aurora 1.4 (NIRx Medical Technologies, Berlin, Germany). The probe configuration for this experiment arranged the emitter and detector pairs to result in 48 long separation channels (~30–35 mm apart) and 8 short separation channels (7 mm apart). The fNIRS cap was set up by locating the Cz position (27) at the midpoint between the nasion and inion and the periauricular points and visually inspected for alignment along the midsagittal plane (Figure 2). Precise localization of each fNIRS channel was determined using a 3D digitizer (Polhemus Patriot, USA) with a custom interface for accurate placement of the digitization stylus in the optode holder. Channels were then grouped into regions of interest using the digitized points that were converted to Montreal Neurological Institute using AtlasViewer (28) and then Brodmann labels using the Allen Human Brain Atlas (29) and the Yale BioImage Suite Package web application (30). Regions of interest included the PFC, PMC, SMC, and PPC. For breakdown on the number of channels that contributed to each region for each participant see Supplementary Table 3. Channels over cortical stroke lesions were identified using individual MRIs and removed from further analysis.

#### Cognitive Status

The Montreal Cognitive Assessment (MoCA) (31) was used to assess global cognition. It assesses a range of cognitive constructs such as: visuoconstructional skills, memory, attention and concentration, executive function, language, conceptual thinking, calculations, and orientation. It has excellent inter-rater reliability (32).

#### Motor Status

The lower extremity portion of the Fugl-Meyer Assessment (FMLE) (33) was used to evaluate motor impairment after stroke. It has excellent inter- (34) and intra-rater (35) reliability, and is a recommended outcome measure for individuals living after stroke (36).

Both MoCA and FMLE were conducted by a trained physiotherapist either on the same day of or within 7 days of fNIRS testing.

#### Gait Speed

Gait speeds were first calculated for every trial by determining the distance walked during the 30 s trials. An average gait speed was calculated for each condition. A percent change in gait speed between the single- and dual-task conditions was calculated using the following formula:


$$\begin{array}{l} Percent\;Change\;=\;\frac{Dual\;Task\;Gait\;Speed-Single\;Task\;Gait\;Speed}{Single\;Task\;Gait\;Speed}\times \;100 \end{array}$$

With this equation, a positive percent shows an increase in gait speed during the dual-task conditions (i.e., *DT-Easy* and *DT-Hard*) compared to the single task (i.e., *ST-Walk)*. Whereas a negative percentage indicates a decrease in gait speed during dual- compared to single-task.

### Analysis

To assess the impact of secondary tasks on walking performance, paired *t*-tests were conducted between *ST-Walk, DT-Easy*, and *DT-Hard*. A corrected *p* ≤ 0.017 was used to adjust for multiple comparisons.

Preprocessing of fNIRS data was completed using an open source software, HomER2 (37). All HomER2 functions and corresponding parameters are indicated within square brackets. Noisy channels were first removed [enPruneChannels: SNRtresh = 2, dRange = 5e-4 to 1e + 00, SDrange:0–45] and compared with the calibration from the Aurora software. Data were then converted into optical density [hmrIntensity2OD]. Motion artifacts were then identified by using 0.5 s time windows to determine if the signal exceeded either 20 standard deviations above the mean signal for each channel or showed a change >100 times in amplitude [hmrMotionArtifactByChannel: tMotion = 0.5, tMask = 1.0, STDEVthresh = 20.0, AMPthresh = 100.00]. The number of removed channels for each participant can be found in Supplementary Table 4. Motion correction was then applied using a wavelet transformation with a 1.5 interquartile range [hmrMotionCorrectWavelet: iqr = 1.5] (38, 39). A lowpass filter of 0.15 Hz was then applied to the data [hmrBandpassFilt: lpf = 0.15] and converted to hemoglobin concentration using the modified Beer-Lambert equation [hmrOD2Conc: ppf = 6.0] (40, 41). The hemodynamic response was estimated using a general linear model with an ordinary least squares approach (42, 43) and a 0.5 s width and 0.5 s step consecutive gaussian basis function (44). Superficial contributions to the signal were also removed by regressing out the data from the short separation channel that has the highest correlation to each channel (43–47). Any drift within the signal was corrected using a 3rd order polynomial correction (43) [hmrDeconvHRF_DriftSS: trange = −20.0 35.0, glmSolveMethod = 1, idxBasis = 1, paramsBasis = 0.5 0.5, rhoSD_ssThresh = 15.0, flagSSmethod = 1, driftOrder = 3, flagMotionCorrect = 0].

Preprocessed data were exported to a custom Matlab script for baseline corrections (−5–0 s before walking onset). Brain activations during the task were then determine by averaging hemoglobin response amplitudes during the first 20 s of walking and subtracting this average by the average response during the 5 s prior to walking.

To look at the effects of increasing walking complexity on brain activation, the conditions (*ST-Walk, DT-Easy, DT-Hard)* were included as fixed effects within a linear mixed model. Hemisphere (ipsilesional, contralesional) was also included as fixed effects and Participants were added as random effects. Four separate linear mixed models were created for each region of interest (PFC, PMC, SMC, PPC). Both HbO and HbR were assessed. As HbO is more reproducible and stable over time (48), has the highest correlation to fMRI BOLD measures (49), and has shown more changes with walking after a stroke [(12) *current issue*], results will focus on HbO findings and HbR results will only be presented as tables in the Supplementary Material. The statistical package “lme4” within the R Studio software was used to model the data. Interaction effects between fixed variables were assessed and only included if it significantly contributed to the model.

To assess the relationship between changes in brain activation and cognitive or motor status, Pearson's correlations were computed. If data was not normally distributed or contained outliers, Spearman's rank-order correlations were computed instead. Relationships between changes in brain activation (*DT-Easy* minus *ST-Walk* and *DT-Hard* minus *ST-Walk*) and MoCA and FMLE scores were assessed.

All relevant assumptions and diagnostics were checked for each statistical test and appropriate modifications were made and reported when necessary. Given the relatively small sample size with exploratory objectives, we present the data results with a standard alpha of 0.05 in order not to miss potential relationships. We also show the data with a Bonferroni corrected *p* ≤ 0.0125 (0.05/4 models) and Benjamini–Hochberg corrected correlation findings demonstrating the results if we reduce the risk of false positives (type 1 error) with multiple comparison.

To facilitate future discussion and investigations on diversity within neuroscientific findings (50), we conducted exploratory subgroup analyses to explore any possible differences with sex. All brain data across each condition were averaged across sex and effect sizes and confidence intervals of the difference between the two sexes are reported.

## Results

All 20 participants completed the *ST-Walk* and *DT-Hard* condition. One participant was too fatigued to continue and did not complete the *DT-Easy* condition. Participant average demographic and performance data are presented in Table 1. Detailed individual data are presented in Supplementary Table 1.

**Table 1:** Participant demographic and performance data.

	***N*** **= 20**
Age [mean (SD)]	64 (7.6) years
Sex (Female/Male)	7/13
Chronicity [mean (SD)]	82 (67.4) months
Lesion Depth (cortical/subcortical/mixed)	0/17/3
Lesion side (Left/Right)	7/13
Gait Aids [none/walking stick(s)/4-point cane/4-wheeled walker]	12/6/1/1
FM-LE (34 max)	27 (4.9)
MoCA (30 max)	26 (2.6)
*ST-Walk* Gait Speed [mean (SD)]	0.83 (0.349) m/s, range: 0.12–1.39 m/s
*DT-Easy* Gait Speed [mean (SD)]	0.85 (0.358) m/s, range: 0.11–1.46 m/s
Percent change from *ST-Walk*	1.88 (8.918)%;
*t*-test results compared to *ST-Walk*	*t*_(18)_ = −0.689, *p* = 0.50
*DT-Hard* Gait Speed [mean (SD)]	0.745 (0.323) m/s, range: 0.09–1.32 m/s
Percent change from *ST-Walk*	−9.84 (9.412)%
*t*-test results compared to *ST-Walk*	*t*_(19)_ = 4.702, *p* < 0.001^*^
*t*-test results compared to *DT-Easy*	*t*_(18)_ = 3.785, *p* < 0.001^**^

* *Indicates significant difference in gait speed compared to ST-Walk*,

** *indicates significant difference in gait speed compared to DT-Easy*.

### Performance Results

The mean gait speed from the *ST-Walk* to the *DT-Easy* was similar. Individual data showed that ten participants increased their speed [8.70 (5.071)%] while eight participants decreased their speed [−6.40 (5.107)%]; one participant showed no change in gait speed. For *DT-Hard*, there was a significant reduction of about 10% in speed; three participants increased their gait speed [7.25 (1.328)%] while 17 participants decreased their gait speed [−12.86 (6.366)%]. Group averages of gait speed for each condition are shown in Table 1 and individual performance data are shown in Supplementary Table 1.

**Figure 3:**
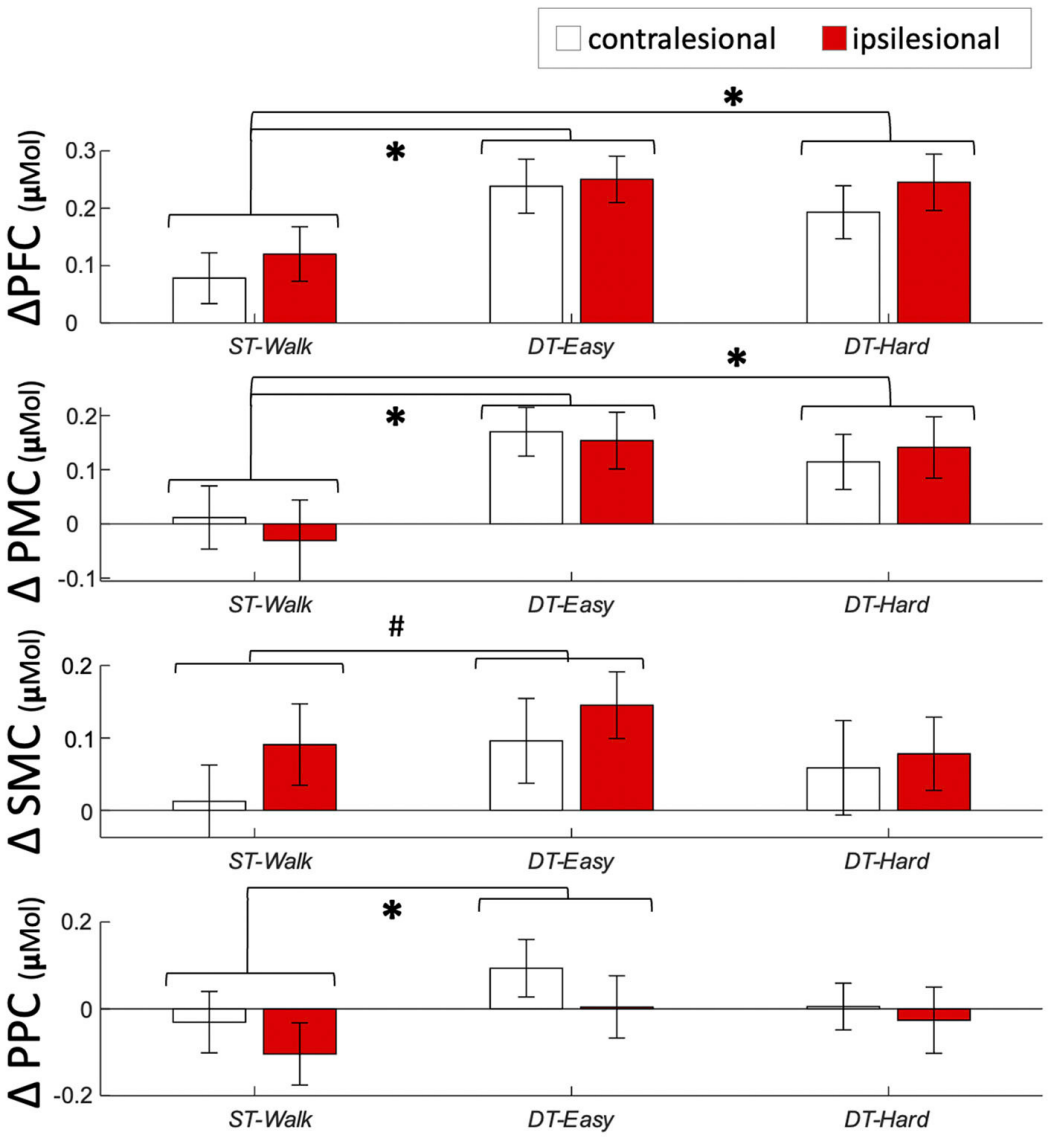
Average and standard error activation during each walking condition. #Indicates a significant difference between conditions with *p* <0.05 ^*^indicates a significant difference between conditions with *p* <0.0125. PFC, prefrontal cortex; PMC, pre-motor cortex; SMC, sensorimotor cortex; PPC, posterior parietal cortex.

Significant effects of walking condition were observed with a significant increase in activation in PFC and PMC during both *DT-Easy* and *DT-Hard* conditions compared to *ST-Walk* (Table 2, Figure 3). A significant increase in PPC activation was found for *DT-Easy* compared to *ST-Walk*. There was an increase in SMC activation for *DT-Easy* with the standard alpha of 0.05 but it did not reach significance with a Bonferroni correction. There was a significant effect of Hemisphere for PFC with the ipsilesional hemisphere overall showing greater activation compared to the contralesional hemisphere. There was also an effect of Hemisphere for PPC with the contralesional hemisphere showing greater activation with the standard alpha, but it did not remain significant with the Bonferroni correction. The inclusion of an interaction term (Hemisphere^*^Condition) did not significantly add to the model and thus was not included in the final model.

**Table 2:** Linear mixed-model results using the model: HbO ~ Condition+Hemisphere + (1|Participant).

**ROI**	**Predictors**	**Estimates**	**Confidence Interval**	***p***	**ICC**	**N_subj_**	**Observations**	**Marginal *R^2^*/Conditional *R^2^***
PFC	(Intercept)	0.076	−0.0001–0.1531	0.051	0.24	20	843	0.043/0.271
	Condition [*DT-Easy*]	0.141	0.0956–0.1872	**<0.001** ^*^				
	Condition [*DT-Hard*]	0.114	0.0693–0.1592	**<0.001** ^*^				
	Hemisphere [ipsi]	0.053	0.0153–0.0903	**0.006** ^*^				
PMC	(Intercept)	−0.008	−0.1063–0.0902	0.872	0.23	20	757	0.035/0.257
	Condition [*DT-Easy*]	0.177	0.1146–0.2391	**<0.001** ^*^				
	Condition [*DT-Hard*]	0.140	0.0787–0.2013	**<0.001** ^*^				
	Hemisphere [ipsi]	−0.009	−0.0598–0.0422	0.735				
SMC	Intercept)	0.031	−0.0652–0.1277	0.526	0.28	20	436	0.009/0.288
	Condition [*DT-Easy*]	0.077	0.0095–0.1448	**0.025**				
	Condition [*DT-Hard*]	0.032	−0.0341–0.098	0.342				
	Hemisphere [ipsi]	0.023	−0.0333–0.0786	0.427				
PPC	(Intercept)	−0.037	−0.1443–0.0699	0.496	0.16	20	568	0.019/0.174
	Condition [*DT-Easy*]	0.128	0.0414–0.2149	**0.004** ^*^				
	Condition [*DT-Hard*]	0.045	−0.0403–0.1297	0.303				
	Hemisphere [ipsi]	−0.076	−0.1471–−0.0053	**0.035**				

*Predictors indicate the fixed effects levels within the variables in the model. Reference levels were ST-Walk for Condition and the contralesional hemisphere for Hemisphere. Estimates indicate the difference between the reference level and the predictor level. Bolded p-values indicate significant differences with an alpha of 0.05*.

* *Indicates significant differences with p ≤ 0.0125 (0.05/4: Bonferroni correction for four models). PFC, prefrontal cortex; PMC, pre-motor cortex; SMC, sensorimotor cortex; PPC, posterior parietal cortex; ipsi, ipsilesional hemisphere*.

**Figure 4:**
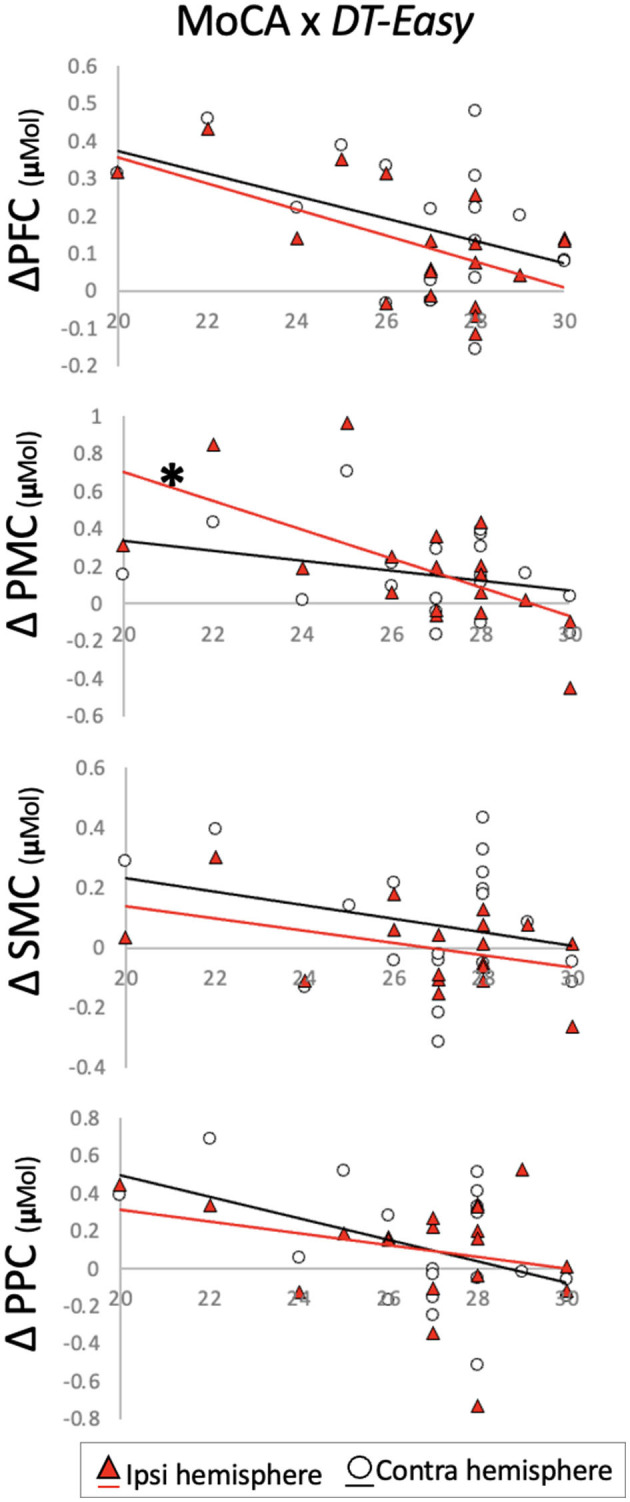
Change in brain activation from (y-axis) *ST-Walk* to *DT-Easy* plotted against Montreal Cognitive Assessment (MoCA) scores (x-axis). ^*^indicates significant correlation after Benjamini–Hochberg correction. PFC, prefrontal cortex; PMC, pre-motor cortex; SMC, sensorimotor cortex; PPC, posterior parietal cortex.

MoCA scores were not normally distributed, and Spearman's rank-order correlations were conducted to account for this. Individual MoCA scores related to brain activation changes during *DT-Easy* and *DT-Hard*. This relationship was only observed in ipsilesional PMC when looking at changes with the easy dual-task. Specifically, higher MoCA scores (i.e., higher cognitive status) related to less increase in brain activation during the dual-task walking conditions compared to walking only (Table 3, Figure 4).

**Table 3:** Spearman's rank-order correlation results between cognitive status (MoCA) and changes in brain activation (ΔHbO).

	**Contralesional**			**Ipsilesional**		
	***r***	***p***	***CI***	***r***	***p***	***CI***
**MoCA ×ΔHbO (*DT-Easy–ST-Walk*)**						
PFC	−0.316	0.187	−0.674–0161	−0.453	0.051	−0.753–−0.001
PMC	−0.212	0.383	−0.608–0.268	**−0.605**	0.006^*^	**−0.831–−0.207**
SMC	−0.115	0.640	−0.541–0.358	−0.215	0.392	−0.620–0.280
PPC	−0.324	0.176	−0.679–−0.152	−0.130	0.596	−0.552–0.345
**MoCA ×ΔHbO (*DT-Hard–ST-Walk*)**						
PFC	0.033	0.890	−0.416–0.469	−0.126	0.598	−0.538–0.336
PMC	−0.252	0.284	−0.625–0.214	**−0.443**	**0.050**	**−0.741–−0.001**
SMC	−0.007	0.977	−0.448–0.437	−0.067	0.793	−0.517–0.413
PPC	−0.256	0.276	−0.627–0.211	−0.425	0.070	−0.737–0.037

*Bolded values indicate a significant relationship with an alpha of 0.05*.

* *Indicates a significant relationship after Benjamini-Hochberg correction using a false discovery rate of 5%. MoCA, Montreal cognitive assessment; PFC, prefrontal cortex; PMC, pre-motor cortex; SMC, sensorimotor cortex; PPC, posterior parietal cortex; CI, confidence intervals*.

**Figure 5:**
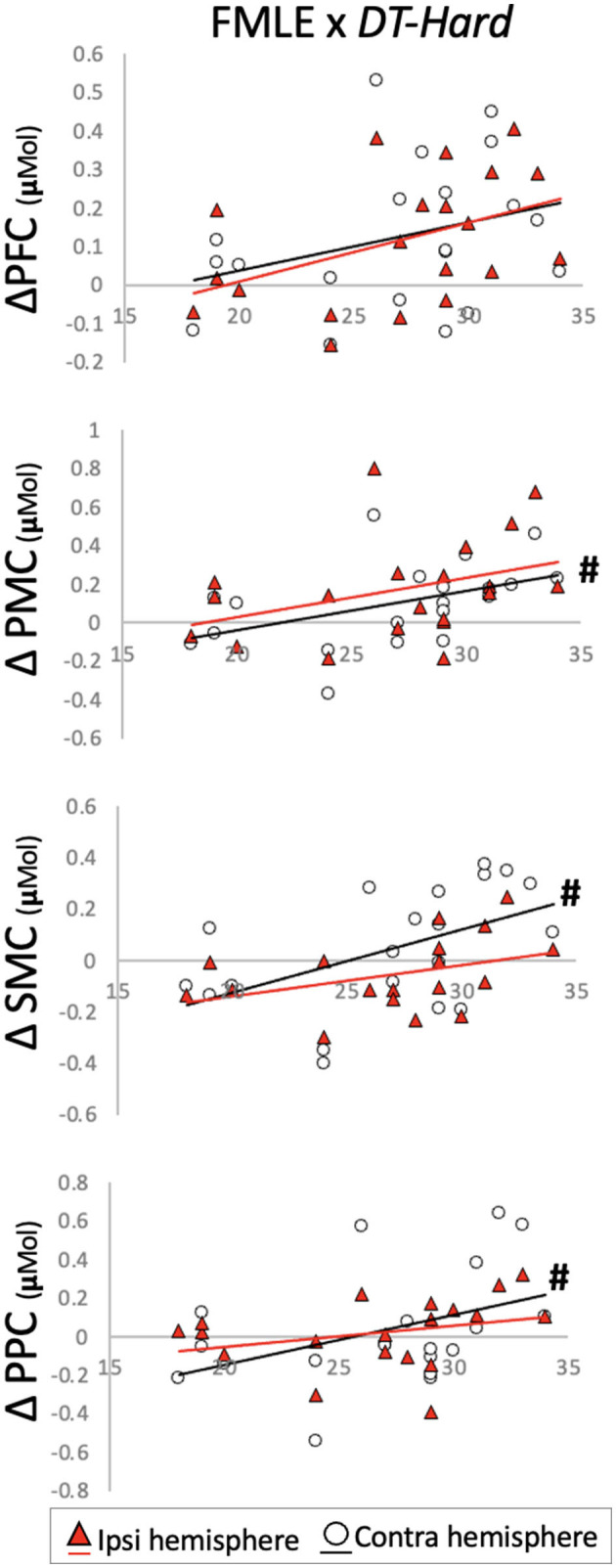
Change in brain activation from (y-axis) *ST-Walk* to *DT-Hard* plotted against the Fugl-Meyer Lower Extremity (FMLE) scores (x-axis). #Indicates significant correlation with alpha at 0.05. PFC, prefrontal cortex; PMC, pre-motor cortex; SMC, sensorimotor cortex; PPC, posterior parietal cortex.

Individual FMLE scores related to brain activation changes during the *DT-Hard* condition only. These relationships were observed in contralesional PMC and PPC and in bilateral SMC. Less impairment (i.e., higher FMLE scores) correlated to a greater increase in brain activation from the -*ST-Walk* condition. After correction for multiple comparisons, however, no relationships remained significant (Table 4, Figure 5).

**Table 4:** Correlation results between motor status (FMLE) and changes in brain activation (ΔHbO).

	**Contralesional**			**Ipsilesional**		
	***r***	***p***	***CI***	***r***	***p***	***CI***
**FMLE ×ΔHbO (*DT-Easy–ST-Walk*)**						
PFC	0.202 (Pearson's)	0.408	−0.278–0.601	0.338 (Pearson's)	0.157	−0.137–0.687
PMC	0.245 (Pearson's)	0.313	−0.236–0.629	0.222 (Pearson's)	0.361	−0.258–0.614
SMC	0.293 (Pearson's)	0.223	−0.186–0.660	0.221 (Pearson's)	0.363	−0.259–0.614
PPC	0.350 (Pearson's)	0.142	−0.124–0.694	0.150 (Pearson's)	0.539	−0.326–0.566
**FMLE ×ΔHbO (*DT-Hard–ST-Walk*)**						
PFC	0.314 (Pearson's)	0.178	−0.149–0.664	0.439 (Pearson's)	0.053	−0.004–0.738
PMC	**0.458** (Pearson's)	**0.042**	**−0.019–0.749**	0.369 (Pearson's)	0.110	−0.088–0.697
SMC	**0.501** (Pearson's)	**0.024**	**0.076–0.773**	0.383 (Pearson's)	0.117	−0.102–0.721
PPC	**0.444** (Spearman's)	**0.050**	**0.002–0.741**	0.293 (Pearson's)	0.223	−0.186–0.659

*Bolded values indicate a significant relationship with an alpha of 0.05. Specific tests used to calculate each correlation is indicated in each cell (Pearson's or Spearman's). MoCA, Montreal cognitive assessment; PFC, prefrontal cortex; PMC, pre-motor cortex; SMC, sensorimotor cortex; PPC, posterior parietal cortex; CI, confidence intervals*.

Changes in brain activation between *ST-walk* and *DT-Easy* for contralesionally PPC were not normally distributed and Spearman's rank-order correlations were conducted when determining relationships with the MoCA or FMLE. One outlier was also found for ipsilesional SMC and PPC and was removed before correlation analysis.

Table 5 shows the brain activation data separated by sex. In general, mean values show a trend toward brain activation levels being greater in male vs. female participants. Medium effect sizes were primarily found between males and females for the *ST-Walk* condition. A range of small to large effect sizes were calculated for the *DT-Easy* condition and only small effect sizes were calculated for the *DT-Hard* condition. Confidence intervals for all brain regions crossed zero, indicating greater sample sizes are needed to find differences between sexes for regions and tasks with medium to large effect sizes.

**Table 5:** Subgroup comparisons of brain activation across sex.

**Condition**	**Brain region**	**Hemisphere**	**Sex**	**Mean (μMol)**	**Standard deviation**	**Cohen's D effect size**	**Confidence interval** **for difference**	
							**(Lower)**	**(upper)**
Normal-Paced walking only	PFC	Contralesional	M	0.07178369	0.27934182	0.14	−0.24634	0.33
			F	0.03018897	0.31674526			
		Ipsilesional	M	0.19716005	0.32694938	0.54^*^	−0.14282	0.49
			F	0.02151173	0.31600656			
	PMC	Contralesional	M	0.08923783	0.32387479	0.62^*^	−0.13023	0.58
			F	−0.1348908	0.4225276			
		Ipsilesional	M	0.0861968	0.39444564	0.74^*^	−0.12	0.84
			F	−0.2741962	0.63516105			
	SMC	Contralesional	M	0.04130268	0.35206055	0.58^*^	−0.17	0.65
			F	−0.1979405	0.52008632			
		Ipsilesional	M	−0.0365042	0.45123856	0.49	−0.27	0.81
			F	−0.3043831	0.69884776			
	PPC	Contralesional	M	0.09889941	0.27168257	0.68^*^	−0.19	0.54
			F	−0.0149505	0.50376289			
		Ipsilesional	M	0.15544131	0.28927676	0.45	−0.08	0.47
			F	−0.0149505	0.50376289			
Easy dual-task walking	PFC	Contralesional	M	0.24485421	0.31053309	0.32	−0.20	0.39
			F	0.14840391	0.28755408			
		Ipsilesional	M	0.31178713	0.3145218	0.70^*^	−0.08	0.50
			F	0.10506293	0.25672112			
	PMC	Contralesional	M	0.21906506	0.32172424	0.46	−0.17	0.48
			F	0.06807605	0.34936946			
		Ipsilesional	M	0.18171021	0.40187634	0.20	−0.32	0.49
			F	0.09740352	0.43240129			
	SMC	Contralesional	M	0.14690479	0.37161914	0.33	−0.25	0.52
			F	0.01644651	0.42570316			
		Ipsilesional	M	0.11049131	0.35049604	0.71^*^	−0.1	0.85
			F	−0.2470696	0.71529799			
	PPC	Contralesional	M	0.20093851	0.2833426	0.86^**^	−0.03	0.54
			F	−0.0534338	0.31521377			
		Ipsilesional	M	0.1327923	0.317935	−0.26	−0.47	0.28
			F	0.2324031	0.48817104			
Hard dual-task walking	PFC	Contralesional	M	0.1657589	0.31174549	−0.11	−0.32	0.26
			F	0.1985413	0.26337855			
		Ipsilesional	M	0.26139715	0.35911724	0.08	−0.30	0.36
			F	0.23380659	0.29324388			
	PMC	Contralesional	M	0.1273051	0.36497392	0.07	−0.32	0.37
			F	0.10396049	0.32686567			
		Ipsilesional	M	0.16413493	0.46608942	0.18	−0.35	0.51
			F	0.08752888	0.36566191			
	SMC	Contralesional	M	0.00555024	0.34336865	0.09	−0.35	0.43
			F	−0.0291145	0.48530385			
		Ipsilesional	M	−0.0661285	0.43405664	0.02	−0.47	0.49
			F	−0.0737485	0.59779231			
	PPC	Contralesional	M	0.10505835	0.35842542	0.17	−0.28	0.41
			F	0.04380797	0.34171478			
		Ipsilesional	M	0.11402394	0.36281258	0.05	−0.31	0.34
			F	0.09832902	0.26367109			

* *Indicates medium effect sizes*,

** *Indicates large effect sizes*.

## Discussion

We found increased PFC, PMC, and PPC activation with dual-task walking as we hypothesized; however, the magnitude of activation did not further increase with increasing complexity of dual-task walking. We did not observe any increase of SMC activation with increasing task complexity. We also showed relationships between the amount of activation increase with walking complexity and individuals' cognitive status. Individuals with higher MoCA scores had lower increases in brain activity when walking while completing an easy dual-task compared to walking on its own.

### Dual-Task Walking Involves Executive Function, Motor Planning, and Sensorimotor Integration Areas

Increases in executive function and motor planning regions were observed for both dual-task conditions. Increases in the executive function area (i.e., PFC) during dual-task walking are well-known and have been observed in several populations (14, 51–53). Increases in PMC, however, are less investigated and have only been documented once using fNIRS in the stroke population (54). Liu et al. (54) looked at PFC, PMC, and supplementary motor area activation during dual-task walking in individuals >6 months post-stroke. Their participants were on average over 10 years younger (51 vs. 64 years) and had greater walking ability (minimum comfortable walking speed of 0.58 vs. 0.12 m/s, and no gait aids vs. 7 people using gait aids) compared to the current study. Despite these demographic differences, they similarly found increases in bilateral PMC with cognitive dual-task walking compared to walking only. Together, these two studies suggest an important need for ongoing motor planning and preparation during complex walking after stroke.

Greater activity in PPC was observed for the easier dual-task condition only. With an additional task, a greater amount of sensorimotor integration would be needed to perform the task. It is possible that the increased complexity of the harder dual-task required resources to be allocated away from PPC to meet the demands of other regions for task completion. Increases in other regions would then be observed. We, however, did not find any increases in activation from the easier to harder dual-task, which may suggest that resources were being directed to subcortical regions such as the cerebellum (4). Further research is needed to test this hypothesis. Alternatively, the nature of the easier dual-task may explain the activation of PPC. PPC is known to be involved in rhythmic, beat-based timing (55) and for the easier dual-task, participants typically repeated the word in a rhythmic manner. This beat-like vocalization was not specifically documented in this study and further work is needed to understand the differential role of PPC in dual-tasking.

### A Brain Activation and Gait Performance Trade-Off

Interestingly, the increase in dual-task complexity did not result in a further increase in activation for the brain regions measured. While previous literature suggests an increase in brain activity with increasing task complexity (56, 57), only one previous study had investigated different difficulties of dual-task walking in individuals post-stroke (58). Hermand et al. assessed PFC activation during walking only compared to walking while completing a 1-back and a 2-back cognitive test. No differences in brain activation were found between any walking condition. Significant decreases in gait speed were seen in both dual-task conditions and the harder dual-task resulted in greater errors on the secondary task. Thus, their lack of PFC increase suggests that a limit was reached, which then came at a detriment to performance. In the current study, we only showed a decrease in gait speed with the harder dual-task and not the easier dual-task despite seeing increases in PFC and PMC activity for both conditions and no differences between the two dual-tasks. Along the same lines of the study by Hermand et al. (58), our results could be explained by a brain activation and performance trade off. For the easier dual-task, increases in brain activity were needed to maintain gait performance, however, with the harder dual-task, a limit may have been reached and this came at a cost to gait performance. This is in line with the Capacity Limit Theory (7).

### Brain Activation Changes Relating to Cognitive and Motor Status

For the easier dual-task, it appears that individuals with better general cognitive status (i.e., higher MoCA score) do not activate brain regions as much as those with lower MoCA scores. This negative relationship is observed in the regions involved in executive function (PFC) and motor planning/preparation (PMC), and integration (PPC) areas, with the strongest correlation in the motor planning/preparation region. Along similar lines, previous work has shown that individuals who show greater intelligence also show more efficient brain activity (59, 60). This may suggest that individuals who have a higher cognitive status may be more efficient (i.e., less additional resources required) at performing easy dual-task walking. Alternatively, the easy verbal task may simply not require any additional resources for individuals with higher cognitive status. Thus, combining walking with the easy verbal task does not elevate brain activity more than just walking alone. With a harder dual-task, however, the relationship is not as strong. With an alpha of 0.05, a significant moderate correlation was observed for ipsilesional PMC only. It's possible that the cognitive demand of the harder verbal task requires additional resources from all individuals. In fact, Neubauer and Fink (61) conducted a review to assess the relationship between intelligence and neural efficiency. They concluded that those with higher intelligence show great efficiency with subjectively easy tasks whereas brain activity during harder tasks was not as efficient and possibly even greater in those with higher intelligence.

On the other hand, with an alpha of 0.05, relationships with an individual's Fugl-Meyer score showed that those with higher motor status activated motor regions (PMC, SMC, PPC) to a greater extent. This may be because they have a greater capacity to increase these brain regions and increase it to a greater extent to mitigate the reductions in gait speed during the harder dual-task. To further explore this, we ran a Pearson's correlation between individual's motor status and their percent change in gait speed for the hard dual-task; they were not correlated (*r*^2^ = 0.003, *p* = 0.74). Alternatively, the greater increase may simply be due to their actual gait speeds during the hard dual-task: motor status and dual-task gait speed were correlated (*r*^2^ = 0.443, *p* = 0.001). This suggests that these individuals simply activate motor regions more because they are able to walk faster.

### Limitations

The study results are limited to the specific dual-tasks measured. Previous studies have utilized numerous secondary tasks with walking and have found differing results (21, 22, 54, 58, 62, 63). While some have suggested that verbal tasks are not recommended with fNIRS due to the higher potential of motion artifacts created (53), the secondary tasks that were chosen for this study were intentional. Yang et al. (64) assessed the psychometric properties of different secondary tasks with walking and showed the greatest reliability with using a verbal fluency task. Reliability was an important factor to consider for the purposes of future interventional or longitudinal research and the clinical impact of these results. In addition, pilot tests and visual inspection of the data did not show any motion artifacts related to verbal responses, especially after pre-processing.

Our results are also limited to the small sample size and inter-subject variability. To account for this, results were presented with both an alpha of 0.05 and with corrections for multiple comparisons. The large range in comfortable walking speed and functional ambulation also likely contributes to the variability in functional brain activation. With a small sample size, subgroup analyses were not possible. Future work should utilize our results to determine appropriate sample sizes for subgroup analyses.

## Conclusions

This is the first study to investigate frontal to parietal brain activations during real-time walking with a secondary task and relate it to cognitive and motor status post-stroke. An increase in walking complexity resulted in an increase in executive function, motor preparation/planning and sensorimotor integration areas. Increasing difficulty of the dual-task walking did not result in further increase in brain activation. In comparison to walking only, individuals with lower cognitive status required larger increases in executive function, motor preparation/planning, and sensorimotor integration areas during the easier dual-task walking. These findings provide further insight on the mechanisms of complex walking post-stroke. Future work should aim to assess longitudinal changes in brain activations during complex walking after stroke and determine if training can optimize dual-task performance and change functional brain activations.

## Data Availability Statement

The original contributions presented in the study are included in the article/Supplementary Material, further inquiries can be directed to the corresponding author.

## Ethics Statement

The studies involving human participants were reviewed and approved by Clinical Research Ethics Board, University of British Columbia. The patients/participants provided their written informed consent to participate in this study.

## Author Contributions

SL and JE conceived the questions. SL, SP, and C-lY collected the data. SL analyzed the data and prepared the first draft of the manuscript. All authors contributed to the study design and edited the manuscript. All authors contributed to the article and approved the submitted version.

## Funding

The authors would like to acknowledge the funding agencies that help supported this research: University of British Columbia 4-Year Fellowship (SL), Canadian Institute of Health Research (Fellowship for SL and SP; Foundation Grant FND143340 to JE), the Michael Smith Foundation for Health Research (SP), the Canada Research Chairs Program (JE, TL-A, and LB), and the Heart and Stroke Foundation Partnership for Stroke Recovery (Post-doc award to Cl-Y). The funders had no role in study design, data collection, analysis, or preparation of the manuscript.

## Conflict of Interest

The authors declare that the research was conducted in the absence of any commercial or financial relationships that could be construed as a potential conflict of interest.

## Publisher's Note

All claims expressed in this article are solely those of the authors and do not necessarily represent those of their affiliated organizations, or those of the publisher, the editors and the reviewers. Any product that may be evaluated in this article, or claim that may be made by its manufacturer, is not guaranteed or endorsed by the publisher.

## References

[1] Yang L Lam F Huang M He C Pang M. Dual-task mobility among individuals with chronic stroke: changes in cognitive-motor interference patterns and relationship to difficulty level of mobility and cognitive tasks. Euro J Phys Rehabil Med. (2017) 54:526–35. 10.23736/S1973-9087.17.04773-6 28949119

[2] Yang YR Chen YC Lee CS Cheng SJ Wang RY. Dual-task-related gait changes in individuals with stroke. Gait Posture. (2007) 25:185–90. 10.1016/j.gaitpost.2006.03.007 16650766

[3] Tsang CSL Pang MYC. Association of subsequent falls with evidence of dual-task interference while walking in community-dwelling individuals after stroke. Clin Rehabil. (2020) 34:971–80. 10.1177/0269215520923700 32460556

[4] Leone C Feys P Moumdjian L D'Amico E Zappia M Patti F. Cognitive-motor dual-task interference: a systematic review of neural correlates. Neurosci Biobehav Rev. (2017) 75:348–60. 10.1016/j.neubiorev.2017.01.010 28104413

[5] Ruthruff E Pashler HE Klaassen A. Processing bottlenecks in dual-task performance: structural limitation or strategic postponement? Psychon Bull Rev. (2001) 8:73–80. 10.3758/BF03196141 11340869

[6] Wickens CD. Multiple resources and mental workload. Hum Fact. (2008) 50:449–55. 10.1518/001872008X288394 18689052

[7] Tombu M Jolicœur P. A central capacity sharing model of dual-task performance. J Exp Psychol Hum Percept Perform. (2003) 29:3–18. 10.1037/0096-1523.29.1.3 12669744

[8] Pashler H. Dual-task interference in simple tasks: data and theory. Psychol Bull. (1994) 116:220–44. 10.1037/0033-2909.116.2.220 7972591

[9] Mcleod P. A dual task response modality effect: support for multiprocessor models of attention. Q J Exp Psychol. (1977) 29:651–67. 10.1080/14640747708400639

[10] Lim SB Eng JJ. Increased sensorimotor cortex activation with decreased motor performance during functional upper extremity tasks poststroke. J Neurol Phys Ther. (2019) 43:141–50. 10.1097/NPT.0000000000000277 31136449

[11] Udina C Avtzi S Durduran T Holtzer R Rosso AL Castellano-Tejedor C. Functional near-infrared spectroscopy to study cerebral hemodynamics in older adults during cognitive and motor tasks: a review. Front Aging Neurosci. (2020) 11:367. 10.3389/fnagi.2019.00367 32038224 PMC6985209

[12] Lim SB. Shedding light on the brain-characterizing functional brain activation during simple and complex walking after stroke (Ph. D. thesis). University of British Columbia, Vancouver, BC, Canada (2021).

[13] Kahya M Moon S Ranchet M Vukas RR Lyons KE Pahwa R. Brain activity during dual task gait and balance in aging and age-related neurodegenerative conditions: a systematic review. Exp Gerontol. (2019) 128:110756. 10.1016/j.exger.2019.110756 31648005 PMC6876748

[14] Lim SB Louie DR Peters S Liu-Ambrose T Boyd LA Eng JJ. Brain activity during real-time walking and with walking interventions after stroke: a systematic review. J Neuroeng Rehabil. (2021) 18:1–19. 10.1186/s12984-020-00797-w 33451346 PMC7811232

[15] Blumen HM Holtzer R Brown LL Gazes Y Verghese J. Behavioral and neural correlates of imagined walking and walking-while-talking in the elderly. Hum Brain Mapp. (2014) 35:4090–104. 10.1002/hbm.22461 24522972 PMC4106989

[16] Van Impe A Coxon JP Goble DJ Wenderoth N Swinnen SP. Age-related changes in brain activation underlying single- and dual-task performance: visuomanual drawing and mental arithmetic. Neuropsychologia. (2011) 49:2400–9. 10.1016/j.neuropsychologia.2011.04.016 21536055

[17] Gazes Y Rakitin BC Steffener J Habeck C Butterfield B Ghez C. Performance degradation and altered cerebral activation during dual performance: evidence for a bottom-up attentional system. Behav Brain Res. (2010) 210:229–39. 10.1016/j.bbr.2010.02.036 20188768 PMC3531229

[18] Johnson AN Shinohara M. Corticomuscular coherence with and without additional task in the elderly. J Appl Physiol. (2012) 112:970–81. 10.1152/japplphysiol.01079.2011 22223451

[19] Collette F Olivier L Van Der Linden M Laureys S Delfiore G Luxen A. Involvement of both prefrontal and inferior parietal cortex in dual-task performance. Cogn Brain Res. (2005) 24:237–51. 10.1016/j.cogbrainres.2005.01.023 15993762

[20] Rémy F Wenderoth N Lipkens K Swinnen SP. Dual-task interference during initial learning of a new motor task results from competition for the same brain areas. Neuropsychologia. (2010) 48:2517–27. 10.1016/j.neuropsychologia.2010.04.026 20434467

[21] Chatterjee SA Fox EJ Daly JJ Rose DK Wu SS Christou EA. Interpreting prefrontal recruitment during walking after stroke: influence of individual differences in mobility and cognitive function. Front Hum Neurosci. (2019) 13:194. 10.3389/fnhum.2019.00194 31316360 PMC6611435

[22] Hawkins KA Fox EJ Daly JJ Rose DK Christou EA McGuirk TE. Prefrontal over-activation during walking in people with mobility deficits: interpretation and functional implications. Hum Mov Sci. (2018) 59:46–55. 10.1016/j.humov.2018.03.010 29604488 PMC5988641

[23] Newkirk LA Kim JM Thompson JM Tinklenberg JR Yesavage JA Taylor JL. Validation of a 26-point telephone version of the mini-mental state examination. J Geriatr Psychiatry Neurol. (2004) 17:81–7. 10.1177/0891988704264534 15157348

[24] Folstein MF Folstein SE McHugh PR. “Mini-mental state”. A practical method for grading the cognitive state of patients for the clinician. J Psychiatr Res. (1975) 12:189–98. 10.1016/0022-3956(75)90026-6 1202204

[25] Borkowski JG Benton AL Spreen O. Word fluency and brain damage. Neuropsychologia. (1967) 5:135–40. 10.1016/0028-3932(67)90015-2

[26] Peirce J Gray JR Simpson S Macaskill M Höchenberger R Sogo H. PsychoPy2 : experiments in behavior made easy. Behav Res Methods. (2019) 51:195–203. 10.3758/s13428-018-01193-y 30734206 PMC6420413

[27] Jurcak V Tsuzuki D Dan I. 10/20, 10/10, and 10/5 systems revisited: their validity as relative head-surface-based positioning systems. Neuroimage. (2007) 34:1600–11. 10.1016/j.neuroimage.2006.09.024 17207640

[28] Aasted CM Yücel MA Cooper RJ Dubb J Tsuzuki D Becerra L. Anatomical guidance for functional near-infrared spectroscopy: AtlasViewer tutorial. Neurophotonics. (2015) 2:020801. 10.1117/1.NPh.2.2.020801 26157991 PMC4478785

[29] Hawrylycz MJ Lein ES Guillozet-bongaarts AL Shen EH Ng L Miller JA. An anatomically comprehensive atlas of the adult human brain transcriptome. Nature. (2012) 489:391–9. 10.1038/nature11405 22996553 PMC4243026

[30] Lacadie C Fulbright RK Arora J Constable R Papademetris X. Brodmann areas defined in MNI space using a new tracing tool in BioImage suite. In: 14th Annual Meeting of the Organization for Human Brain Mapping (Melbourne, VIC). (2008).

[31] Nasreddine Z Phillips N Bedirian V Charbonneau S Whitehead V Collin I. The montreal cognitive assessment, MoCA: a brief screening tool for mild cognitive impairment. J Am Geriatr Soc. (2005) 53:695–9. 10.1111/j.1532-5415.2005.53221.x 15817019

[32] Cumming TB Lowe D Linden T Bernhardt J. The AVERT MoCA data: scoring reliability in a large multicenter trial. Assessment. (2020) 27:976–81. 10.1177/1073191118771516 29877095

[33] Fugl-Meyer AR Jääsk, ö L Leyman I Olsson S Steglind S. The post-stroke hemiplegic patient. 1. A method for evaluation of physical performance. Scand J Rehabil Med. (1975) 7:13–31. 1135616

[34] Duncan P Propst M Nelson S. Reliability of the Fugl-Meyer assessment of sensorimotor recovery following cerebrovascular accident. Phys Ther. (1983) 63:1606–10. 10.1093/ptj/63.10.1606 6622535

[35] Sullivan KJ Tilson JK Cen SY Rose DK Hershberg J Correa A. Fugl-Meyer assessment of sensorimotor function standardized training procedure for clinical practice and clinical trials. Stroke. (2011) 42:427–32. 10.1161/STROKEAHA.110.592766 21164120

[36] Kwakkel G Lannin NA Borschmann K English C Ali M Churilov L. Standardized measurement of sensorimotor recovery in stroke trials: consensus-based core recommendations from the stroke recovery and rehabilitation roundtable. Int J Stroke. (2017) 12:451–61. 10.1177/1747493017711813 28697709

[37] Huppert TJ Diamond SG Franceschini MA Boas DA. HomER: a review of time-series analysis methods for near-infrared spectroscopy of the brain. Appl Opt. (2009) 48:D280–98. 10.1364/AO.48.00D280 19340120 PMC2761652

[38] Molavi B Dumont GA. Wavelet-based motion artifact removal for functional near-infrared spectroscopy. Physiol Meas. (2012) 33:259–70. 10.1088/0967-3334/33/2/259 22273765

[39] Hocke LM Oni IK Duszynski CC Corrigan AV Frederick BD Dunn JF. Automated processing of fNIRS data-A visual guide to the pitfalls and consequences. Algorithms. (2018) 11:67. 10.3390/a11050067 30906511 PMC6428450

[40] Kocsis L Herman P Eke A. The modified Beer-Lambert law revisited. Phys Med Biol. (2006) 51:N91–8. 10.1088/0031-9155/51/5/N02 16481677

[41] Sassaroli A Fantini S. Comment on the modified Beer-Lambert law for scattering media. Phys Med Biol. (2004) 49:N255–7. 10.1088/0031-9155/49/14/N07 15357206

[42] Ye JC Tak S Jang KE Jung J Jang J. NIRS-SPM: statistical parametric mapping for near-infrared spectroscopy. NeuroImage. (2009) 44:428–47. 10.1016/j.neuroimage.2008.08.036 18848897

[43] Aasted CM Yücel MA Steele SC Peng K Boas DA Becerra L. Frontal lobe hemodynamic responses to painful stimulation: A potential brain marker of nociception. PLoS ONE (2016) 11:e0165226. 10.1371/journal.pone.0165226 27806119 PMC5091745

[44] Yücel MA Selb J Aasted CM Lin PY Borsook D Becerra L. Mayer waves reduce the accuracy of estimated hemodynamic response functions in functional near-infrared spectroscopy. Biomed Opt Express. (2016) 7:3078–88. 10.1364/BOE.7.003078 27570699 PMC4986815

[45] Gagnon L Cooper RJ Yücel MA Perdue KL Greve DN Boas DA. Short separation channel location impacts the performance of short channel regression in NIRS. Neuroimage. (2012) 59:2518–28. 10.1016/j.neuroimage.2011.08.095 21945793 PMC3254723

[46] Yücel MA Aasted CM Petkov MP Borsook D Boas DA Becerra L. Specificity of hemodynamic brain responses to painful stimuli: a functional near-infrared spectroscopy study. Sci Rep. (2015) 5:9469. 10.1038/srep09469 25820289 PMC4377554

[47] Yücel MA Selb J Aasted CM Petkov MP Becerra L Borsook D. Short separation regression improves statistical significance and better localizes the hemodynamic response obtained by near-infrared spectroscopy for tasks with differing autonomic responses. Neurophotonics. (2015) 2:35005. 10.1117/1.NPh.2.3.035005 26835480 PMC4717232

[48] Plichta MM Herrmann MJ Baehne CG Ehlis A Richter MM Pauli P. Event-related functional near-infrared spectroscopy (fNIRS): are the measurements reliable? Neuroimage. (2006) 31:116–24. 10.1016/j.neuroimage.2005.12.008 16446104

[49] Strangman G Culver JP Thompson JH Boas DA. A quantitative comparison of simultaneous BOLD fMRI and NIRS recordings during functional brain activation. Neuroimage. (2002) 17:719–31. 10.1006/nimg.2002.1227 12377147

[50] Dotson VM Duarte A. The importance of diversity in cognitive neuroscience. Ann N Y Acad Sci. (2020) 1464:181–91. 10.1111/nyas.14268 31663150

[51] Pelicioni PHS Tijsma M Lord SR Menant J. Prefrontal cortical activation measured by fNIRS during walking: effects of age, disease and secondary task. PeerJ. (2019) 7:e6833. 10.7717/peerj.6833 31110922 PMC6501770

[52] Stuart S Vitorio R Morris R Martini DN Fino PC Mancini M. Cortical activity during walking and balance tasks in older adults and in people with Parkinson's disease: a structured review. Maturitas. (2018) 113:53–72. 10.1016/j.maturitas.2018.04.011 29903649 PMC6448561

[53] Vitorio R Stuart S Rochester L Alcock L Pantall A. fNIRS response during walking — artefact or cortical activity? A systematic review. Neurosci Biobehav Rev. (2017) 83:160–72. 10.1016/j.neubiorev.2017.10.002 29017917

[54] Liu YC Yang YR Tsai YA Wang RY Lu CF. Brain activation and gait alteration during cognitive and motor dual task walking in stroke-A functional near-infrared spectroscopy study. IEEE Trans Neural Syst Rehabil Eng. (2018) 26:2416–23. 10.1109/TNSRE.2018.2878045 30371378

[55] Ross JM Iversen JR Balasubramaniam R. The role of posterior parietal cortex in beat-based timing perception: a continuous theta burst stimuation study. J Cogn Neurosci. (2018) 30:634–43. 10.1162/jocn_a_01237 29346017

[56] Holper L Biallas M Wolf M. Task complexity relates to activation of cortical motor areas during uni- and bimanual performance: A FUNCTIONAL NIRS study. Neuroimage. (2009) 46:1105–13. 10.1016/j.neuroimage.2009.03.027 19306929

[57] Lopes TM Yasuda CL Campos BM de Balthazar MLF Binder JR Cendes F. Effects of task complexity on activation of language areas in a semantic decision fMRI protocol. Neuropsychologia. (2016) 81:140–8. 10.1016/j.neuropsychologia.2015.12.020 26721760

[58] Hermand E Tapie B Dupuy O Fraser S Compagnat M Salle JY. Prefrontal cortex activation during dual task with increasing cognitive load in subacute stroke patients: a pilot study. Front Aging Neurosci. (2019) 11:160. 10.3389/fnagi.2019.00160 31312136 PMC6614381

[59] Ramchandran K Zeien E Andreasen NC. Distributed neural efficiency: intelligence and age modulate adaptive allocation of resources in the brain. Trends Neurosci Educ. (2019) 15:48–61. 10.1016/j.tine.2019.02.006 31176471

[60] Schultz DH Cole MW. Higher intelligence is associated with less task-related brain network reconfiguration. J Neurosci. (2016) 36:8551–61. 10.1523/JNEUROSCI.0358-16.2016 27535904 PMC4987432

[61] Neubauer AC Fink A. Intelligence and neural efficiency. Neurosci Biobehav Rev. (2009) 33:1004–23. 10.1016/j.neubiorev.2009.04.001 19580915

[62] Al-Yahya E Johansen-Berg H Kischka U Zarei M Cockburn J Dawes H. Prefrontal cortex activation while walking under dual-task conditions in stroke: a multimodal imaging study. Neurorehabil Neural Repair. (2016) 30:591–9. 10.1177/1545968315613864 26493732 PMC5404717

[63] Mori T Takeuchi N Izumi SI. Prefrontal cortex activation during a dual task in patients with stroke. Gait Posture. (2018) 59:193–8. 10.1016/j.gaitpost.2017.09.032 29073516

[64] Yang L He C Pang MYC. Reliability and validity of dual-task mobility assessments in people with chronic stroke. PLoS ONE. (2016) 11:e0147833. 10.1371/journal.pone.0147833 26808662 PMC4726712

