# Supplementary material for: Frontal, Sensorimotor, and Posterior Parietal Regions Are Involved in Dual-Task Walking After Stroke
Source: Front Neurol. 2022 Jun 22;13:904145. doi: 10.3389/fneur.2022.904145 (PMC9256933; doi:10.3389/fneur.2022.904145)
Supplement: Supplementary file 1 [file Data_Sheet_1.docx]

Supplementary Table 1. Individual participant demographic data

| Participant ID | Age | Sex | Stroke chronicity (months) | Lesion side and depth | FMLE (/34) | MoCA (/30) | *ST-Walk* gait speed (m/s) | *DT-Easy* gait speed (m/s) | *DT-Hard* gait speed (m/s) |
| --- | --- | --- | --- | --- | --- | --- | --- | --- | --- |
|  |  |  |  |  |  |  |  |  |  |
| S01 | 63 | M | 21 | R subcortical | 31 | 28 | 1.19 | 1.26 | 1.13 |
| S02 | 58 | M | 6 | R subcortical | 27 | 28 | 0.87 | 0.98 | 0.85 |
| S03 | 67 | M | 46 | R subcortical | 29 | 27 | 0.8 | 0.79 | 0.68 |
| S04 | 61 | F | 15 | L mixed | 29 | 24 | 0.66 | DNC | 0.62 |
| S05 | 58 | F | 21 | R mixed | 19 | 24 | 0.14 | 0.15 | 0.16 |
| S06 | 49 | F | 55 | R subcortical | 30 | 27 | 0.72 | 0.70 | 0.60 |
| S07 | 50 | F | 13 | R subcortical | 33 | 25 | 1.36 | 1.15 | 1.32 |
| S08 | 67 | M | 115 | L subcortical | 28 | 28 | 1.39 | 1.46 | 1.29 |
| S09 | 62 | F | 230 | L subcortical | 26 | 22 | 0.57 | 0.67 | 0.62 |
| S10 | 54 | M | 12 | R subcortical | 20 | 30 | 0.78 | 0.77 | 0.68 |
| S11 | 73 | M | 162 | R subcortical | 32 | 28 | 1.11 | 0.98 | 0.92 |
| S12 | 67 | M | 67 | R subcortical | 27 | 26 | 0.95 | 1.00 | 1.01 |
| S13 | 72 | M | 144 | R mixed | 24 | 29 | 0.54 | 0.61 | 0.50 |
| S14 | 63 | M | 38 | R subcortical | 29 | 30 | 0.9 | 0.85 | 0.77 |
| S15 | 69 | F | 159 | L subcortical | 18 | 26 | 0.18 | 0.11 | 0.09 |
| S16 | 72 | M | 126 | R unknown | 19 | 28 | 0.51 | 0.55 | 0.46 |
| S17 | 69 | M | 50 | L subcortical | 29 | 27 | 0.85 | 0.78 | 0.70 |
| S18 | 74 | F | 128 | R subcortical | 31 | 28 | 0.71 | 0.79 | 0.58 |
| S19 | 71 | M | 52 | L subcortical | 24 | 27 | 1.19 | 1.21 | 0.97 |
| S20 | 71 | M | 179 | L unknown | 34 | 20 | 1.16 | 1.50 | 0.94 |

FMLE=Fugl-Meyer Lower Extremity, MoCA=Montreal Cognitive Assessment, DNC= did not complete

Supplementary Table 2. HbR results

| ROI | Predictors | Estimates | Confidence Interval | p | ICC | N_subj_ | Observations | Marginal R^2^ / Conditional R^2^ |
| --- | --- | --- | --- | --- | --- | --- | --- | --- |
| PFC | (Intercept) | -0.005137 | -0.032889 – 0.022614 | 0.717 | 0.11 | 20 | 843 | 0.022 / 0.132 |
|  | Condition [DT-Easy] | 0.031749 | 0.009309 – 0.054189 | **0.006 *** |  |  |  |  |
|  | Condition [DT-Hard] | -0.012900 | -0.034919 – 0.009118 | 0.251 |  |  |  |  |
|  | Hemisphere [ipsi] | 0.021524 | 0.003185 – 0.039863 | **0.021** |  |  |  |  |
| PMC | (Intercept) | -0.031845 | -0.073187 – 0.009498 | 0.131 | 0.14 | 20 | 757 | 0.007 / 0.142 |
|  | Condition [DT-Easy] | 0.030318 | -0.002268 – 0.062904 | 0.068 |  |  |  |  |
|  | Condition [DT-Hard] | -0.001763 | -0.033854 – 0.030327 | 0.914 |  |  |  |  |
|  | Hemisphere [ipsi] | -0.017687 | -0.044367 – 0.008993 | 0.194 |  |  |  |  |
| SMC | (Intercept) | -0.050268 | -0.097752 – -0.002783 | **0.038** | 0.12 | 20 | 436 | 0.009 / 0.124 |
|  | Condition [DT-Easy] | 0.042142 | -0.002960 – 0.087243 | 0.067 |  |  |  |  |
|  | Condition [DT-Hard] | 0.000423 | -0.043840 – 0.044686 | 0.985 |  |  |  |  |
|  | Hemisphere [ipsi] | -0.004737 | -0.041883 – 0.032409 | 0.803 |  |  |  |  |
| PPC | (Intercept) | -0.088923 | -0.151094 – -0.026752 | **0.005 *** | 0.08 | 20 | 568 | 0.004 / 0.087 |
|  | Condition [DT-Easy] | 0.038265 | -0.021628 – 0.098158 | 0.210 |  |  |  |  |
|  | Condition [DT-Hard] | 0.025742 | -0.032981 – 0.084464 | 0.390 |  |  |  |  |
|  | Hemisphere [ipsi] | -0.022019 | -0.070901 – 0.026862 | 0.377 |  |  |  |  |

Predictors indicate the fixed effects levels within the variables in the model. Reference levels were *ST-Walk* for Condition and the contralesional hemisphere for Hemisphere. Estimates indicate the difference between the reference level and the predictor level. Bolded p-values indicate significant differences with an alpha of 0.05. * indicates significant differences with p≤0.0125 (0.05 /4: Bonferroni correction for four models). PFC=prefrontal cortex, PMC=premotor cortex, SMC=sensorimotor cortex, PPC=posterior parietal cortex, ipsi=ipsilesional hemisphere

**Supplementary Table 3. Number of channels contributing to each region of interest for each participant**

|  | **# PFC channels** | | **# PMC channels** | | **# SMC channels** | | **# PPC channels** | |
| --- | --- | --- | --- | --- | --- | --- | --- | --- |
|  | ipsi | contra | ipsi | contra | ipsi | contra | ipsi | contra |
| S01 | 8 | 5 | 7 | 7 | 4 | 3 | 6 | 4 |
| S02 | 8 | 8 | 6 | 7 | 3 | 4 | 6 | 4 |
| S03 | 8 | 7 | 6 | 8 | 3 | 4 | 6 | 4 |
| S04 | 6 | 9 | 5 | 6 | 5 | 3 | 5 | 6 |
| S05 | 2 | 7 | 2 | 6 | 0 | 4 | 2 | 5 |
| S06 | 8 | 5 | 5 | 10 | 1 | 2 | 5 | 5 |
| S07 | 7 | 5 | 7 | 6 | 4 | 3 | 3 | 4 |
| S08 | 6 | 7 | 7 | 7 | 4 | 5 | 4 | 4 |
| S09 | 6 | 8 | 7 | 6 | 4 | 5 | 5 | 5 |
| S10 | 8 | 7 | 6 | 6 | 4 | 5 | 5 | 5 |
| S11 | 7 | 8 | 6 | 8 | 5 | 3 | 3 | 5 |
| S12 | 8 | 6 | 7 | 8 | 4 | 4 | 5 | 4 |
| S13 | 7 | 6 | 6 | 6 | 2 | 3 | 5 | 5 |
| S14 | 8 | 8 | 6 | 3 | 5 | 1 | 5 | 4 |
| S15 | 8 | 8 | 8 | 5 | 3 | 5 | 5 | 4 |
| S16 | 8 | 8 | 5 | 8 | 3 | 2 | 7 | 5 |
| S17 | 7 | 9 | 7 | 6 | 3 | 2 | 5 | 7 |
| S18 | 10 | 5 | 5 | 9 | 4 | 4 | 3 | 2 |
| S19 | 7 | 9 | 5 | 6 | 3 | 5 | 4 | 4 |
| S20 | 8 | 8 | 5 | 5 | 4 | 5 | 6 | 5 |

PFC=prefrontal cortex, PMC=premotor cortex, SMC=sensorimotor cortex, PPC=posterior parietal cortex, ipsi=ipsilesional hemisphere, contra=contralesional hemisphere

**Supplementary Table 4. Number of channels excluded from analysis**

|  | **# PFC channels** | | **# PMC channels** | | **# SMC channels** | | **# PPC channels** | | **total channels excluded** |
| --- | --- | --- | --- | --- | --- | --- | --- | --- | --- |
|  | ipsi | contra | ipsi | contra | ipsi | contra | ipsi | contra |  |
|  | excluded | excluded | excluded | excluded | excluded | excluded | excluded | excluded |  |
| S01 | 0 | 2 | 0 | 0 | 0 | 0 | 0 | 0 | **2** |
| S02 | 0 | 0 | 0 | 0 | 0 | 0 | 0 | 0 | **0** |
| S03 | 0 | 0 | 0 | 0 | 0 | 0 | 0 | 0 | **0** |
| S04 | 0 | 0 | 0 | 0 | 1 | 0 | 0 | 0 | **1** |
| S05 | 5 | 0 | 5 | 0 | 4 | 0 | 3 | 1 | **18** |
| S06 | 0 | 0 | 2 | 0 | 2 | 0 | 0 | 1 | **5** |
| S07 | 0 | 1 | 0 | 1 | 0 | 2 | 2 | 1 | **7** |
| S08 | 0 | 0 | 0 | 0 | 0 | 0 | 2 | 0 | **2** |
| S09 | 0 | 0 | 0 | 0 | 0 | 0 | 0 | 0 | **0** |
| S10 | 0 | 0 | 0 | 0 | 0 | 0 | 0 | 0 | **0** |
| S11 | 0 | 0 | 1 | 0 | 0 | 0 | 0 | 0 | **1** |
| S12 | 0 | 0 | 0 | 0 | 0 | 0 | 0 | 0 | **0** |
| S13 | 1 | 0 | 1 | 1 | 1 | 0 | 2 | 0 | **6** |
| S14 | 0 | 0 | 0 | 2 | 0 | 4 | 0 | 0 | **6** |
| S15 | 0 | 0 | 0 | 0 | 0 | 0 | 0 | 0 | **0** |
| S16 | 0 | 0 | 0 | 0 | 0 | 0 | 0 | 0 | **0** |
| S17 | 0 | 0 | 0 | 0 | 0 | 0 | 0 | 0 | **0** |
| S18 | 0 | 0 | 0 | 0 | 0 | 0 | 1 | 3 | **4** |
| S19 | 0 | 0 | 0 | 0 | 1 | 1 | 0 | 1 | **3** |
| S20 | 0 | 0 | 0 | 0 | 0 | 0 | 0 | 0 | **0** |

PFC=prefrontal cortex, PMC=premotor cortex, SMC=sensorimotor cortex, PPC=posterior parietal cortex, ipsi=ipsilesional hemisphere, contra=contralesional hemisphere


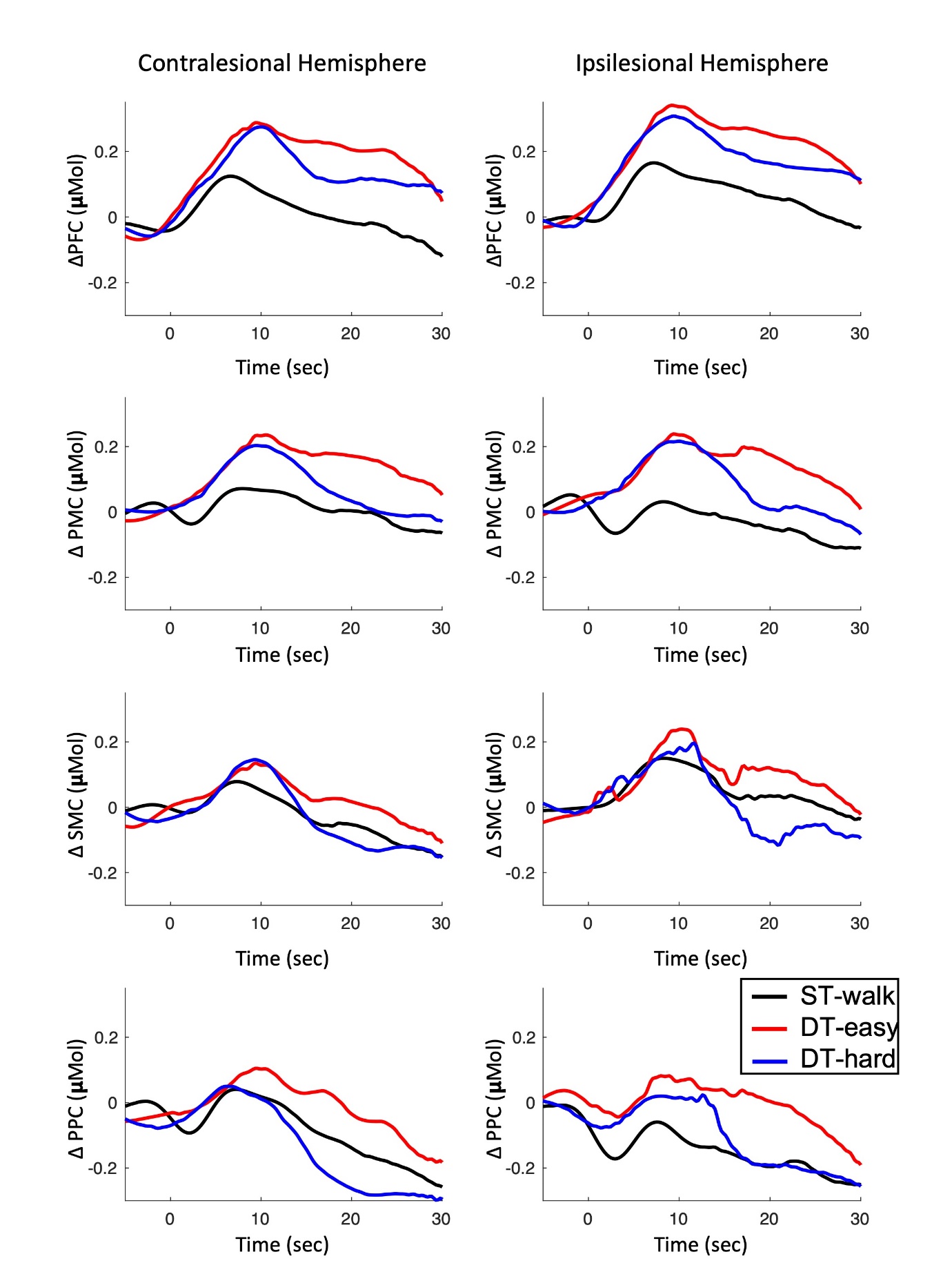


**Supplementary Figure 1. fNIRS waveform for each region of interest**. Each walking condition is plotted in different colours on a single subplot.
